# 

**Published:** 1851-09

**Authors:** 


WHOLE SERIES—VOL. VIII, NO. III.

VOL. IV.] SEPTEMBER, 1851. [No. 3.

### THE NORTH-WESTERN MEDICAL AND SURGICAL JOURNAL.

PUBLISHED EVERY OTHER MONTH.

AT TWO DOLLARS PER ANNUM IN ADVANCE.

EDITED BY JOHN EVANS, M.D.,

PROFESSOR OF OBSTETRICS &C. IN RUSH MED. COL.; MEMBER OF THE AM. MED. ASSOCIATION; ONE OF THE PHYSICIANS TO THE ILL. GEN. HOSPITAL; FORMERLY SUP'T OF THE IND. HOSPITAL FOR THE INSANE, ETC., ETC.

CHICAGO AND INDIANAPOLIS:
PRINTED BY JAS. J. LANGDON,
161 Lake street, Chicago.
1851.

ENLARGED SERIES—VOL. VI, NO. III.

